# Long-Term Spatiotemporal Stability and Dynamic Changes in the Haemoparasite Community of Bank Voles (*Myodes glareolus*) in NE Poland

**DOI:** 10.1007/s00248-014-0390-9

**Published:** 2014-03-07

**Authors:** Anna Bajer, Renata Welc-Falęciak, Małgorzata Bednarska, Mohammed Alsarraf, Jolanta Behnke-Borowczyk, Edward Siński, Jerzy M. Behnke

**Affiliations:** 1 Department of Parasitology, Institute of Zoology, Faculty of Biology, University of Warsaw, 1 Miecznikowa Street, 02-096 Warsaw, Poland; 2 Department of Forest Phytopathology, Faculty of Forestry, Poznań University of Life Sciences, Poznań, Poland; 3 School of Life Sciences, University of Nottingham, University Park, Nottingham, NG7 2RD UK

## Abstract

Long-term field studies on parasite communities are rare but provide a powerful insight into the ecological and evolutionary processes shaping host–parasite interactions. The aim of our study was to identify the principal factors regulating long-term trends in the haemoparasite communities of bank voles, and to this end, we sampled three semi-isolated populations of bank voles (*n* = 880) in 1999, 2002, 2006 and 2010 in the Mazury lake district region of NE Poland. Overall, 90.8 % of the bank voles harboured at least one of the species of haemoparasites studied. Whilst overall prevalence (all species combined) did not vary significantly between the surveys, different temporal changes were detected among voles in each of the three sites. In voles from Urwitałt, prevalence increased consistently with successive surveys, whereas in Tałty, the peak years were 2002 and 2006, and in Pilchy, prevalence oscillated without a clear pattern. Across the study, bank voles harboured a mean of 1.75 ± 0.034 haemoparasite species, and species richness remained stable with no significant between-year fluctuations or trends. However, each of the five constituent species/genera showed a different pattern of spatio-temporal changes. The overall prevalence of *Babesia microti* was 4.9 %, but this varied significantly between years peaking in 2006 and declining again by 2010. For *Bartonella* spp., overall prevalence was 38.7 %, and this varied with year of study, but the temporal pattern of changes differed among the three sites. The overall prevalence of *Haemobartonella* (*Mycoplasma*) was 68.3 % with an increase in prevalence with year of study in all three sites. *Hepatozoon erhardovae* had an overall prevalence of 46.8 % but showed a marked reduction with each successive year of the study, and this was consistent in all three sites. The overall prevalence of *Trypanosoma evotomys* was 15.4 % varying significantly between sites, but showing temporal stability. While overall prevalence of all haemoparasites combined and species richness remained stable over the period of study, among the five haemoparasites, the pattern of spatiotemporal changes in prevalence and abundance of infections differed depending on parasite species. For some genera, host age was shown to play an important role, but a significant effect of host sex was found only for *Haemobartonella* spp.

## Introduction

Long-term field studies on parasite communities provide a powerful insight into ecological and evolutionary processes shaping host–parasite interactions over time. Small mammals, especially rodents, are good model hosts for such studies because their populations are abundant, heterogenous and highly dependent on environmental factors, including food availability and climatic conditions [22, 32], and are subject to the impact of parasites (Cowpox virus—[23]; hantavirus—[43]). Haemoparasites in particular are likely to be an important source of selective pressure on hosts because they are often associated with pathogenicity (e.g. acute babesiosis, trypanosomiasis [14, 52, 57]), and hence, resistance/tolerance of such infections confers enormous fitness benefits [34, 35, 45]. Thus, in host populations challenged intensively by haemoparasites, and yet showing high population densities over years, we may expect relatively stable/repeatable and defined relationships in their host–parasite interactions as a consequence of the long periods of co-evolution and resultant stabilisation [45].

The high heterogeneity and the dynamic between- and within-year variation of rodent populations allow investigation of the relative contribution of a range of quantifiable intrinsic and extrinsic factors underlying some of the dominant patterns of variation in parasitic infections observed in the field [5, 11, 12]. Each rodent community can be regarded as comprising a set of different functional subgroups including, for example, settled, territorial adults of both sexes and mobile juveniles, which may differ in their exposure and susceptibility to infection [48]. The role of these subgroups as hosts for different haemoparasites is not known nor the precise contribution of unpredictable external factors (i.e. temperature and humidity) that create complex and, perhaps at times, unique temporary combinations of environmental/climatic effects. Because of the still limited number of systematic long-term ecological studies in naturally infected hosts, most published studies having been confined to periods of four or fewer years [8, 9, 17, 31, 39, 58, 73, 74, 76, 82], little is known about the longer term dynamics of different haemoparasites in host populations and their subgroups [69].

In addition to their importance as model populations in ecological studies of host–parasite interactions, small mammals, including rodents, also play a crucial role in the epidemiology of zoonotic infections and those transmissible to domestic livestock [28, 29, 36, 50]. Many important vectors, including juvenile ticks and fleas, depend on rodents as hosts at some stage during their life cycle [15, 16, 20, 61, 78], and these in turn provide a source of pathogens that can be transmitted via the vectors to humans and livestock. Species for which they act as reservoirs in Europe for example include microparasites such as *Bartonella* spp., *Anaplasma phagocytophilum*, *Neoehrlichia mikurensis*, *Borrelia burgdorferi* and *Babesia microti*, all of which are also important vector-borne pathogens (VBP) of humans and domestic animals [18, 20, 21, 58–60, 65, 66, 78, 81].

Research on the parasite communities of wild rodents and ticks has been conducted in N.E. Poland in the Mazury lake district region, continuously to date since 1997. These investigations have focussed over the last 16 years on two rodent communities: woodland with the model species being *Myodes glareolus* and *Apodemus flavicollis* and fallow land with *Microtus arvalis*, *Microtus agrestis* and *Microtus oeconomus* [9, 59, 60, 65, 66, 78, 81]. Different parasite communities have been studied in detail, including ticks foraging on different rodent hosts [61, 65, 66, 78], haemoparasite communities [9, 58] and helminths [7, 10–13]. In 2005, preliminary genetic characterisation of the bank voles from three woodland sites was carried out, based on six microsatellite loci, which showed a distinct population structure and linked some parasite burdens with host MHC DRB diversity [44].

Although a range of contemporary molecular techniques has been used to study microparasite species in rodents in Poland [59, 60, 78, 81], the ecological characteristics/significance of the H/P relationships have received less attention, and to date, their precise roles in the functional haemoparasite community have been relatively neglected. Therefore, to attain a better understanding of the underlying processes, in 1999, we initiated a long-term project on the haemoparasite community of bank voles in three semi-isolated populations, which we studied subsequently at 3–4-year intervals over a period of 11 years (1999–2010). We set out to verify previously reported molecular and ecological findings/patterns, building on these in the context of long-term records.

The aim of the current study therefore was to evaluate the influence of four quantifiable ecological factors (host sex and age, and site and year of study) on the characteristic haemoparasite community of the bank voles over a period of 11 years, with a particular focus on the latter two factors, i.e. on spatiotemporal variation. We predicted that the effect of intrinsic factors (e.g. age and sex) would be consistent and repeatedly observable in successive surveys, whether influencing parasitaemia significantly or not, showing little between-year variation in magnitude of the effect due to co-evolution of the hosts and parasites involved. Haemoparasites are VBP and in short-lived host species, such as bank vole, we would expect a significant increase in the prevalence of VBP with host age, as the probability of being infested with ticks or fleas carrying VBP increases also with host age. However, there are increasing numbers of reports of congenital infections (vertical transmission) with VBPs, including bacterial and protozoan parasites [24, 42, 56, 64]. In these cases, we would expect the pattern of age-depended relationships to be reversed with the highest prevalence in the youngest hosts. Host immune responses to each of the haemoparasites differ, and where host-protective immunity is generated, we would also expect both prevalence and abundance of infection to decline in the oldest age class [68]. We predicted that extrinsic factors in general, but temporal effects in particular, would have a greater influence resulting in distinct between-survey dynamics. Annual variation in infection levels is highly dependent on climatic conditions (which can vary markedly from year to year in the same geographic location) and apart from the expected seasonal cycles in prevalence and abundance, exact climatic conditions (e.g. severity of winters, dry versus wet summers etc.) are largely ‘unpredictable’ for both hosts and parasites. However, because of their proximity to one another and similarity in their ecology [13], we expected between-site/between-population differences in haemoparasite community to remain relatively stable over a period of 11 years. From the epidemiological point of view, such long-term studies on the spatio-temporal dynamics of human pathogens/microparasites in rodent population are rare in the literature, but cumulatively, they are likely to provide a better understanding of the dynamics of VBP and the associated diseases they are responsible for in humans and domestic livestock.

## Materials and Methods

### Study Sites

The study sites have been described comprehensively by Behnke et al. [11, 13]. They are located in Mazury in the north eastern corner of Poland, in the vicinity of Jezioro (Lake) Śniardwy and the towns of Mikołajki, Ryn and Pisz. Site 1 is referred to as Urwitałt (N 53 × 48.153, EO 21 × 39.784), site 2 as Tałty (N 53 × 53.644, EO 21 × 33.049) and site 3 as Pilchy (N 53 × 42.228, EO 21 × 48.499) after nearby settlements.

### Collection of Bank Voles

The methods used for trapping rodents, and for sampling and processing of trapped animals, have all been fully described by Behnke et al. [11, 13]. All the sampled voles were culled, since internal helminth parasites were also quantified (to be published elsewhere), so this was a cross-sectional study in each of the 4 years when surveys were undertaken. Age categories were established as described by Behnke et al. [13], and here, age class 1 includes young juvenile animals not yet mature enough to be reproductively active, age class 2 young adults and age class 3 the oldest animals in the study. The breakdown of voles sampled by year, site and age for the first two surveys (1999 and 2002) is given in previous papers on helminth communities [11, 12] and is shown for the entire period in Table 1.

**Table 1:** No of voles sampled in successive surveys, by site, and host age and sex

Site	Year	Sex	Age class			Totals	
			1	2	3	Row	Site and year
Urwitałt	1999	Male	0	13	5	18	
		Female	2	6	9	17	35
	2002	Male	8	13	15	36	
		Female	6	12	13	31	67
	2006	Male	12	38	15	65	
		Female	15	14	21	50	115
	2010	Male	8	33	8	49	
		Female	9	13	19	41	90
	Total males	28	97	43	168		
	Total females	32	45	62	139		
	Total combined sexes	60	142	105	307		
Tałty	1999	Male	3	13	4	20	
		Female	8	8	5	21	41
	2002	Male	16	15	8	39	
		Female	7	17	10	34	73
	2006	Male	16	13	15	44	
		Female	18	4	19	41	85
	2010	Male	13	14	26	53	
		Female	12	9	22	43	96
	Total males	48	55	53	156		
	Total females	45	38	56	139		
	Total sexes combined	93	93	109	295		
Pilchy	1999	Males	10	10	4	24	
		Females	6	12	5	23	47
	2002	Males	11	14	11	36	
		Females	8	13	16	37	73
	2006	Males	21	14	19	54	
		Females	24	5	17	46	100
	2010	Males	9	7	8	24	
		Females	9	6	19	34	58
	Total males	51	45	42	138		
	Total females	47	36	57	140		
	Total sexes combined	98	81	99	278		
Total by year	1999	Males	13	36	13	62	
		Females	16	26	19	61	
		Both sexes	29	62	32	123	
	2002	Males	35	42	34	111	
		Females	21	42	39	102	
		Both sexes	56	84	73	213	
	2006	Males	49	65	49	163	
		Females	57	23	57	137	
		Both sexes	106	88	106	300	
	2010	Males	30	54	42	126	
		Females	30	28	60	118	
		Both sexes	60	82	102	244	
Total by sex	Males		127	197	138	462	
	Females		124	119	175	418	
	Both sexes		251	316	313	880	

### Detection of Haemoparasites

For this project, five parasites [*B. microti*, *Bartonella* spp., *Hepatozoon erhardovae, Trypanosoma evotomys* and *Haemobartonella* (*Mycoplasma*) spp.] were monitored in surveys conducted in 1999, 2002, 2006 and 2010 in the three study sites, and at the same time of the year, from mid-August to mid-September, when rodent populations peak in this region of Europe and to avoid the known seasonal influences on their haemoparasite communities [9, 58, 76].

### Blood Collection and DNA Extraction

Thin blood smears were prepared from drops of blood taken from the heart or tail tip. Blood smears were air-dried, fixed in absolute methanol and stained for 1 h in Giemsa stain in buffer at pH 7.2. Each smear was examined under oil immersion (×1,000 magnification). Parasites were counted in 200 fields of vision. Microscopical observation of stained blood smears was used as the only detection method for studies in 1999 and 2002.

In 2006 and 2010, in addition to blood smears, molecular techniques were used also for the detection of *Babesia* and *Bartonella* (in all samples) and for species identification in the case of *Hepatozoon and Trypanosoma* infections (only in samples positive by microscopical observation) [3, 78, 79, 81]. From the culled animals, 200 ml of whole blood were collected into 0.001 M EDTA and frozen at a temperature of −20 °C. When individuals were found dead in the trap, the whole heart was isolated and homogenised in 400 ml of 0.001 M EDTA. For this group of animals, blood smears were not obtained, and diagnosis of *Babesia and Bartonella* infections was carried out exclusively on the basis of PCR.

Genomic DNA was extracted from whole blood or heart homogenates using AxyPrep MiniPrep Blood kit (AxyGen, USA) and stored at a temperature of −20 °C. The extracted DNA was subjected to specific PCRs as described in detail in Welc-Falęciak et al. [77, 78] and Alsarraf [3]. The primers and cycling conditions used in this study are listed in Table 2. PCR products were subjected to electrophoresis on a 1.5 % agarose gel, stained with Midori Green stain (Nippon Genetics GmbH) and sequenced by a private company (Genomed S.A., Poland).

**Table 2:** Nucleotide sequences and annealing temperature of the primers used for polymerase chain reaction (PCR)

Species	Gene	Primer	Sequence 5′ → 3′	Annealing temperature (°C)	Fragment size (bp)	Reference
*Babesia*	18S rRNA	GR2 GF2	CCAAAGACTTTGATTTCTCTC G(C/T)(C/T)TTGTAATTGGAATGATGG	60	559	[19]
	18S rRNA	PIROA PIROB	AATACCCAATCCTGACACAGGG TTAAATACGAATGCCCCCAAC	55	437	[4]
*Bartonella*	gltA	BhCS.781p BhCS.1137n	GGGGACCAGCTCATGGTGG AATGCAAAAAGAACAGTAAACA	51	380	[53]
	rpoB	rpoR rpoF	CGCATTATGGTCGTATTTGTCC GCACGATT(C/T)GCATCATCATTTTCC	52	333	[59]
*Hepatozoon*	18S rRNA	Hep1 Hep2	CGCGAAATTACCCAATT CAGACCGGTTACTTTYAGCAG	60	660	[41]
*Trypanosoma*	18S rRNA	TRY927F TRY927R SSU561F SSU561R	CAGAAACGAAACACGGGAG CCTACTGGGCAGCTTGGA TGGGATAACAAAGGAGCA CTGAGACTG TAACCTCAAAGC	58	556	[54, 55]

### Statistical Analysis

Prevalence (percentage of animals infected) was estimated based on microscopical observations and/or PCR results, and values are reported with the 95 % confidence limits, calculated by bespoke software based on the tables of Sokal and Rohlf [70]. Abundance of infection was quantified as the number of infected red blood cells (iRBC) (for *Babesia*, *Bartonella* and *Mycoplasma*) or parasites (for *Trypanosoma* and *Hepatozoon*) in 200 fields of vision at ×1,000 magnification. When samples were only positive by PCR, an intensity of 1 iRBC/1 parasite in 200 fields of vision was implemented into quantitative statistical analysis.

The statistical approach adopted has been documented comprehensively in our earlier publications [6, 11–13]. For analysis of prevalence, we used maximum likelihood techniques based on log linear analysis of contingency tables in the software package SPSS (version 16.0.1, SPSS, Inc., Chicago, IL, USA). Initially, full factorial models were fitted, incorporating as factors sex (two levels, males and females), age (three levels), year of study (four levels, each of the four surveys) and site (three levels, the three study sites). Infection was considered as a binary factor (presence/absence of parasite). These explanatory factors were fitted initially to all models that were evaluated. For each level of analysis in turn, beginning with the most complex model, involving all possible main effects and interactions, those combinations that did not contribute significantly to explaining variation in the data were eliminated in a stepwise fashion beginning with the highest level interaction (backward selection procedure). A minimum sufficient model was then obtained, for which the likelihood ratio of *χ* ^2^ was not significant, indicating that the model was sufficient in explaining the data (these values are given in the legends to the figures as relevant). The importance of each term (i.e. interactions involving infection) in the final model was assessed by the probability that its exclusion would alter the model significantly, and these values relating to interactions that included presence/absence of infection are given in the text. The remaining terms in the final model that did not include presence/absence of infection are not given but can be made available from the authors on request.

For analyses of quantitative data, we used general linear models (GLM) with normal errors implemented in R version 2.2.1 (R Core Development Team), and the residuals were checked for approximate Gaussian distribution. When the residuals failed to meet the requirements of Gaussian model, we used generalised linear models with negative binomial or Poisson error structures. Full factorial models that converged satisfactorily were simplified using the STEP procedure and tested for significance using deletion of terms beginning with the highest order interaction by comparing models with or without that interaction. Changes in deviance (DEV) are given for models based on Poisson errors (interpreted by chi^2^); for models based in Gaussian errors, we give F, and for those based on negative binomial errors, the likelihood ratio (LR). Minimum sufficient models were then fitted (all significant interactions and main effects plus any main effects that featured in interactions), and the process was repeated to obtain values for changes in deviance, test statistics and probabilities. The degree of aggregation in the data was calculated by the index of discrepancy (*D*) as described by Poulin [62] and the index of dispersion (*I*, variance to mean ratio). Frequency distributions of individual taxa were also tested for goodness of fit to negative binomial, positive binomial and Poisson models by chi-squared as described by Elliott [30], and the negative binomial exponent k is given as appropriate. Finally, if the data did not meet the assumptions of parametric tests, we employed non-parametric tests (Kruskal–Wallis test and the Mann–Whitney *U* test).

## Results

### Molecular Identification of Parasite Species (2006 and 2010)

#### *Babesia Microti*

*B. microti* was the rarest of the haemoparasites in this study, but ten isolates from the animals assessed in 2006 and one from those in 2010 (two from Urwitałt, two from Tałty and seven from Pilchy) were genotyped by the amplification and sequencing of a 560-bp fragment of the 18S rRNA gene. All isolates were highly similar (>98.5 % of homology) to *B. microti* Jena strain (EF413181), originally obtained from human blood in Germany [40].

#### *Bartonella* spp.

Among 45 genotyped isolates obtained in 2006 and 2010 (17 from Urwitałt, 13 from Tałty and 15 from Pilchy), two species of *Bartonella* were identified: *Bartonella grahamii* (34 isolates) and *Bartonella taylorii* (11 isolates) [3, 81]. Moreover, among these species, further subdivision was evident into three (*B. grahamii*, DQ450357) and five (*B. taylorii*, EU014275 and EU014280) distinct genetic clades with homologies ranging 88.5–99.7 % [3, 81]. Of the 45 genotyped isolates, 6 (13.3 %) were identical to the *B. grahamii* strain associated with human illness and *B. grahamii* was the dominant species in bank voles at all three sites.

#### *Hepatozoon Erhardovae*

Forty-nine *H. erhardovae* isolates obtained in 2010 (18 from Urwitałt, 22 from Tałty and 9 from Pilchy) were genotyped by the amplification and sequencing of a 660-bp fragment of the *18S rRNA* gene. Comparison with GenBank database revealed the occurrence of two very similar *Hepatozoon* genetic variants [99.5–99.8 % sequence homology to BV1 (AY600626) and BV2 (AY600625)], which have been previously reported from bank voles from Spain [27]. Our isolate, most closely related to BV1, was the dominant variant observed in our three bank vole populations (78 % of the isolates from Urwitałt and Pilchy and 68 % from Tałty were our variant of BV1).

**Fig. 1:**
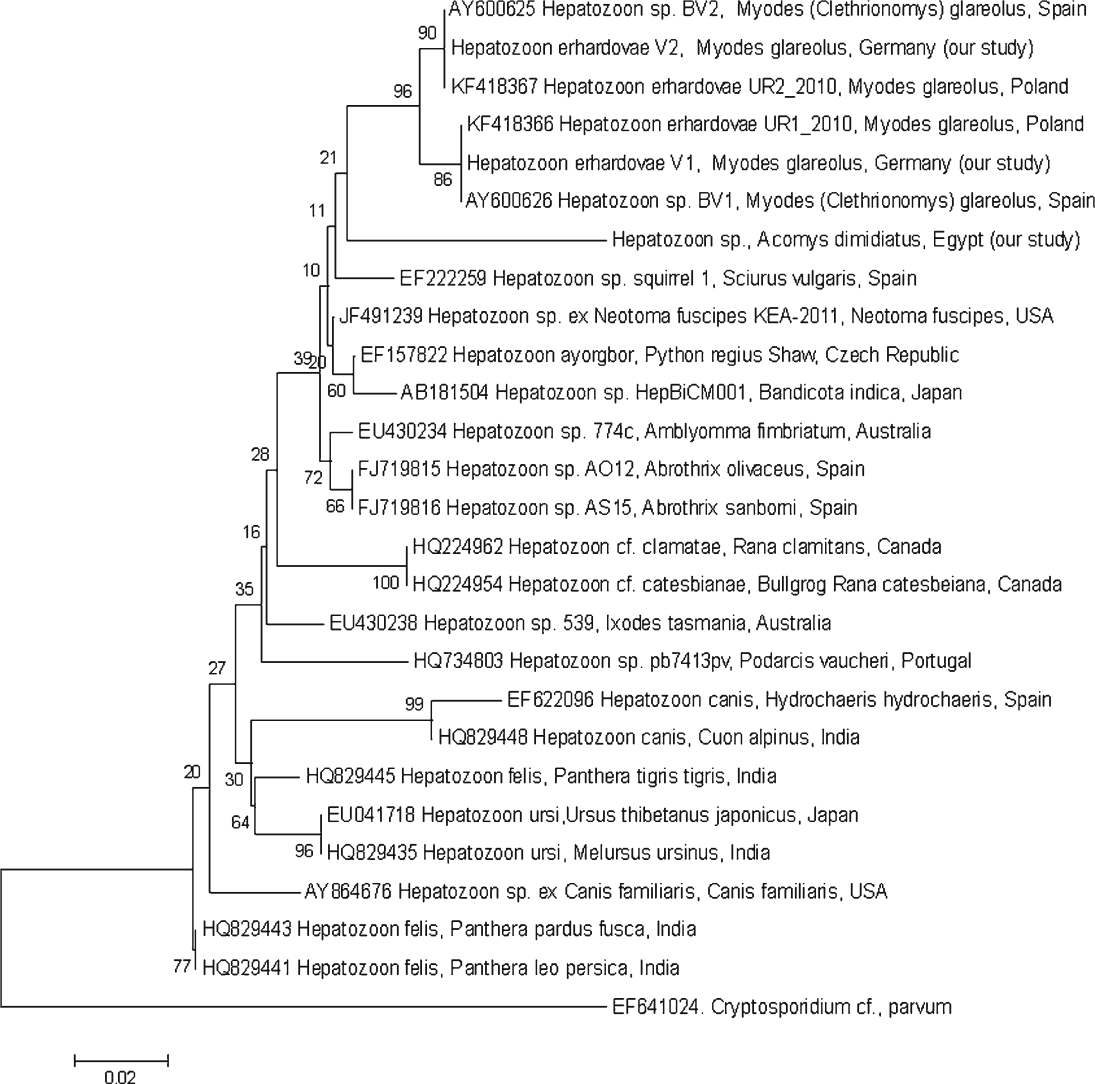
The evolutionary history of *Hepatozoon* based on the fragment of the *18S rRNA* gene was inferred using the neighbour-joining method. The percentage of replicate trees in which the associated taxa clustered together in the bootstrap test (1,000 replicates) are shown next to the branches. The evolutionary distances were computed using the Kimura two-parameter method and are in the units of the number of base substitutions per site. The analysis involved 27 nucleotide sequences. All positions containing gaps and missing data were eliminated. The nucleotide sequence of *Cryptosporidium parvum* was used as an outgroup. Evolutionary analyses were conducted in MEGA5 [72]

Comparison with German *Hepatozoon* isolates derived from bank voles in the Bayern-Munchen region revealed the presence of similar genetic variants (Fig. 1), supporting the occurrence of bank vole specific species—*H. erhardovae* as described by Krampitz [47] in Germany. The unique sequences derived from our isolates have been deposited in GenBank under the species name *H. erhardovae* (accession numbers: KF418366 and KF418367).

#### *Trypanosoma Evotomys*

Twenty-one *Trypanosoma* isolates were obtained in 2010 (12 from Urwitałt, 5 from Tałty and 4 from Pilchy) and genotyped by the amplification and sequencing of a 520-bp fragment of the *18S rRNA* gene [54]. All these isolates were identical and showed 100 % homology with *T. evotomys* (GenBank: AY043356), which has been previously reported from bank voles in the UK [54].

### Prevalence of Haemoparasites

**Fig. 2:**
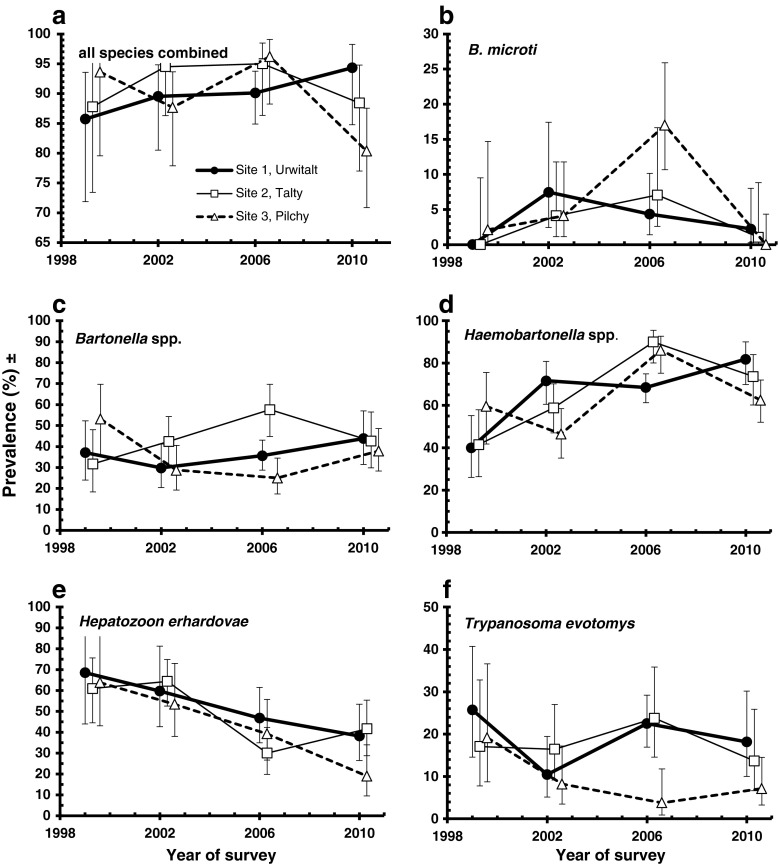
Prevalence of haemoparasites recorded in bank voles at three study sites in NE Poland between 1999 and 2010. For statistical analysis see text

Overall 90.8 % (88.1–92.86) of the 880 voles harboured at least one of the five haemoparasite genera recorded in the study. There was no overall significant change in prevalence with time (Table 3) or between sites; however, prevalence showed different temporal changes in each of the three sites (year × site × presence/absence of haemoparasites *χ*_6_^2^ = 12.9, *P* = 0.044). In voles from Urwitałt prevalence increased consistently survey by survey (from 85.7 % in 1999 to 94.3 % in 2010), whereas in Tałty peak, years were 2002 and 2006, and in Pilchy, prevalence oscillated without a clear pattern (Fig. 2a).

**Table 3:** Prevalence of haemoparasites by year, site, host sex and age class

	Haemoparasites	*B.microti*	*Bartonella* spp.	*Haemobartonella* spp.	*H. erhardovae*	*T. evotomys*
Year						
1999	89.4 (83.76–93.43)	**0.8** (0.12–3.71)	41.5 (34.05–49.15)	48.0 (40.30–55.66)	**64.2** (56.56–71.33)	20.3 (14.73–27.16)
2002	90.6 (87.61–92.7)	**5.2** (3.48–7.54)	33.8 (29.62–38.21)	58.7 (54.18–63.09)	**59.2**(54.65–63.55)	11.7 (9.09–14.97)
2006	93.3 (90.37–95.47)	**9.3** (6.67–12.91)	38.3 (33.36–43.59)	80.0 (75.66–83.72)	**39.6** (34.83–44.63)	17.4 (13.86–21.57)
2010	88.7 (85.33–91.42)	**1.2** (0.52–2.85)	42.0 (37.32–46.74)	74.1 (69.67–78.03)	**35.0** (30.53–39.65)	13.8 (10.82–17.43)
Site						
Urwitałt	90.7 (87.12–93.36)	3.9 (2.26–6.53)	36.9 (31.90–42.23)	69.8 (64.72–74.40)	49.7 (44.38–54.96)	**18.9** (15.11–23.46)
Tałty	91.7 (88.32–94.14)	3.4 (1.91–5.83)	45.4 (40.24–50.64)	69.9 (64.96–74.44)	46.9 (41.71–52.08)	**17.6** (13.98–21.98)
Pilchy	89.8 (86.45–92.47)	7.6 (5.25–10.73)	33.5 (28.82–38.44)	64.7 (59.92–69.25)	43.2 (38.42–48.08)	**8.6** (6.25–11.83)
Sex						
Males	89.2 (84.49–92.79)	5.8 (3.39–9.77)	36.9 (30.65–43.53)	63.5 (56.94–69.56)	48.2 (41.66–54.76)	16.4 (12.00–21.75)
Females	92.5 (88.56–95.14)	3.8 (1.96–7.02)	40.7 (34.58–46.95)	73.7 (67.97–78.78)	45.1 (39.13–51.28)	14.3 (10.45–19.08)
Age^a^						
Class 1	93.6 (90.88–95.58)	4.0 (2.44–6.32)	**47.0** (42.17–51.85)	78.6 (74.54–82.29)	40.0 (35.46–44.69)	**9.4** (6.97–12.54)
Class 2	91.6 (88.11–94.17)	4.7 (2.91–7.60)	**42.4** (37.15–47.80)	63.0 (57.67–68.09)	48.4 (43.02–53.77)	**14.1** (10.76–18.30)
Class 3	87.7 (83.77–90.76)	5.8 (3.69–8.81)	**28.2** (23.61–33.28)	65.7 (60.48–70.52)	50.3 (45.04–55.62)	**21.3** (17.27–25.95)

Significant main effects, not confounded by interactions, are highlighted in bold, but see text for further details

^a^The voles were allocated to three age classes, age class 1 being young juvenile voles, not yet reproductively active, age class 2 were young adult voles and age class 3 the oldest animals in the study. See Materials and Methods for reference to how the animals were allocated to these three classes

### Species Richness

**Fig. 3:**
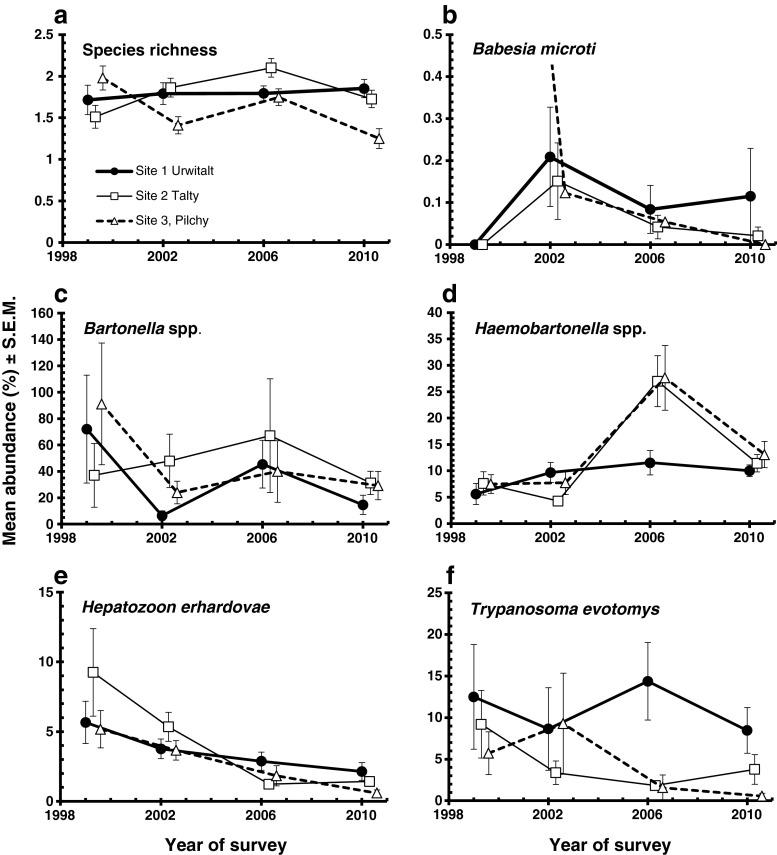
Abundance of haemoparasites recorded in bank voles at three study sites in NE Poland between 1999 and 2010. Data are the number of iRBC or parasites observed/200 fields of vision under ×100 (objective lens) microscopy. In **b**, the value for *B. microti* at Pilchy in 1999 was 1.87, but largely from one heavily infected vole with a value of 88 iRBC/200 fields of vision. For statistical analysis, see text

Across the study, bank voles harboured a mean of 1.75 ± 0.034 haemoparasite species. Species richness differed significantly between sites (Table 4; GLM with Poisson errors, main effect of site, DEV_2_ = 6.04, *P* = 0.05), but the extent of the difference between sites varied between years (Fig. 3a; two-way interaction year × site, DEV_6_ = 13.46, *P* = 0.04). In voles from Urwitałt, mean species richness remained stable across the period of study, whereas in Tałty, it increased slowly, and in Pilchy, it oscillated and was the lowest in 2010 (Fig. 3a). Thus, there was no independent effect of year, and we observed very similar values for overall mean species richness in each of the 4 years of the study (Table 4).

**Table 4:** Abundance of haemoparasite by year, site, sex and age class

	Species richness	*B.microti*	*Bartonella* spp.	*Haemobartonella spp.*	*H. erhardovae*	*T. evotomys*
Year						
1999	1.75 ± 0.088	**0.72** ± 0.715	67.7 ± 22.52	**7.0** ± 1.14	**6.7** ± 1.24	8.8 ± 2.44
2002	1.69 ± 0.068	**0.16** ±0.054	26.7 ±7.67	**7.2** ± 1.02	**4.3** ± 0.49	7.0 ± 2.64
2006	1.87 ± 0.058	**0.06** ±0.026	50.2 ± 16.27	**20.8** ± 2.52	**2.1** ± 0.36	6.9 ± 2.00
2010	1.66 ± 0.066	**0.05** ± 0.043	24.7 ± 5.09	**11.3** ± 0.97	**1.5** ± 0.28	4.7 ± 1.25
Site						
Urwitałt	**1.80** ± 0.058	**0.11** ± 0.047	**30.8** ± 8.54	10.0 ± 1.04	**3.2** ± 0.39	**11.1** ± 2.31
Tałty	**1.83** ± 0.059	**0.06** ± 0.025	**46.4** ± 13.86	13.4 ± 1.57	**3.5** ± 0.56	**3.9** ± 0.91
Pilchy	**1.58** ± 0.059	**0.40** ± 0.343	**42.4** ± 11.69	15.0 ± 2.17	**2.7** ± 0.40	**4.3** ± 1.87
Sex						
Males	1.72 ± 0.049	0.11 ± 0.036	30.3 ± 6.43	**9.6** ± 1.06	3.2 ± 0.41	8.4 ± 1.77
Females	1.78 ± 0.047	0.25 ± 0.220	50.1 ± 12.06	**16.1** ± 1.54	3.0 ± 0.33	4.6 ± 1.01
Age^a^						
Class 1	1.80 ± 0.060	0.06 ± 0.031	**69.3** ± 19.71	**20.5** ± 2.52	2.6 ± 0.43	**6.7** ± 2.33
Class 2	1.74 ± 0.055	0.33 ± 0.284	**34.5** ± 8.56	**8.1** ± 0.80	3.0 ± 0.35	**7.0** ± 1.77
Class 3	1.72 ±0.062	0.11 ± 0.043	**21.4** ± 5.14	**11.3** ± 1.42	3.7 ± 0.55	**6.1** ± 1.45

Significant main effects are highlighted in bold, but see text for further details

^a^The voles were allocated to three age classes, see legend to Table 3 for more information

#### *Babesia Microti*

The overall prevalence of *B. microti* was 4.9 % (3.39–6.96), but prevalence varied significantly between years (Table 3), peaking in 2006, largely as a consequence of the high rate of infection in the population in Pilchy (>15 %) and declining again by 2010 (Fig. 2b; year × presence/absence of *B. microti*, *χ*_3_^2^ = 26.3, *P* < 0.001). There was no independent effect of SITE, but a significant albeit weak complex interaction with other factors, which we did not dissect further, was part of the minimal sufficient model (site × sex × age × presence/absence of *B. microti*, *χ*_4_^2^ = 10.1, *P* = 0.039).

The overall abundance of *B. microti* was 0.18 ± 0.106 iRBC/200 fields of vision, and the infections were highly aggregated (*I* = 46.97, *D* = 0.982, *k* = 0.022 ± 0.000009; for goodness of fit to the negative binomial distribution *χ*_3_^2^ = 13.7, *P* = 0.003). Parametric models based on transformed data showed overdispersed residuals, and those based on negative binomial error structure failed to converge, so analysis was by non-parametric tests. Abundance declined significantly over the four surveys (Table 4, Kruskal–Wallis test *χ*_3_^2^ = 26.2, *P* < 0001) and varied between populations (Table 4, *χ*_2_^2^ = 7.07, *P* = 0.029), but the interaction (Fig. 3b) could not be tested. The high abundance in 1999 at Pilchy and the very wide SEM were largely caused by one animal with 88 iRBC/200 fields. Despite a mean abundance in female voles of more than twice that in males, there were no significant differences in abundance between the sexes or between the age classes (Table 4).

#### *Bartonella* spp.

The overall prevalence of *Bartonella* spp. was 38.7 % (34.7–42.77). Prevalence varied with year of study, but the temporal pattern of change differed between sites (Fig. 2c; year × site × presence/absence of *Bartonella* spp., *χ*_6_^2^ = 20.8, *P* = 0.002), with a peak 2006 in Tałty and a dip in prevalence in Urwitałt in 2002, and in Pilchy in 2002 and 2006, resulting in the greatest discrepancy in infection rates between three populations in 2006 (prevalence of only 25 % in Pilchy versus 58 % in Tałty) (Fig. 2c). Prevalence also declined significantly with increasing host age (Table 3; age × presence/absence of *Bartonella* spp. *χ*_2_^2^ = 25.0, *P* < 0.001).

The overall abundance of *Bartonella* spp. was high (39.7 ± 6.65 iRBC/200 fields) with peaks in 1999 in Urwitałt and Pilchy, populations at both sites showing very similar mean levels of infection across the whole period of the study, and in 2006 among voles from Tałty, which were generally more heavily parasitised (Fig. 3c). Despite these between-year differences and the overall downward drift in the mean abundance of *Bartonella* spp. from 1999 to 2010 (Table 4), there was no significant difference between years (Kruskal–Wallis test *χ*_3_^2^ = 4.96 *P* = 0.18). Statistical models based on Gaussian [on log10(*x* + 1) and square root transformed data] failed to generate normally distributed residuals, and those based on negative binomial error structures would not converge because of the extreme overdispersion of data for this species (*I* = 948.9, *D* = 0.91, and for goodness of fit to negative binomial *χ*_12_^2^ = 13.3 *P* = 0.35 and *k* = 0.0874 ± 0.000025). However, the mean abundance of *Bartonella* spp. declined significantly and markedly (by 69.2 %) with increasing age of voles (Table 4; Kruskal–Wallis test *χ*_2_^2^ = 27.6 *P* < 0.001) and there was a weaker effect of SITE (Table 4; Kruskal–Wallis test *χ*_2_^2^ = 7.3 *P* = 0.026) with overall abundance highest in voles from Tałty and lowest at Urwitałt.

#### *Haemobartonella* (*Mycoplasma*) spp.

**Fig. 4:**
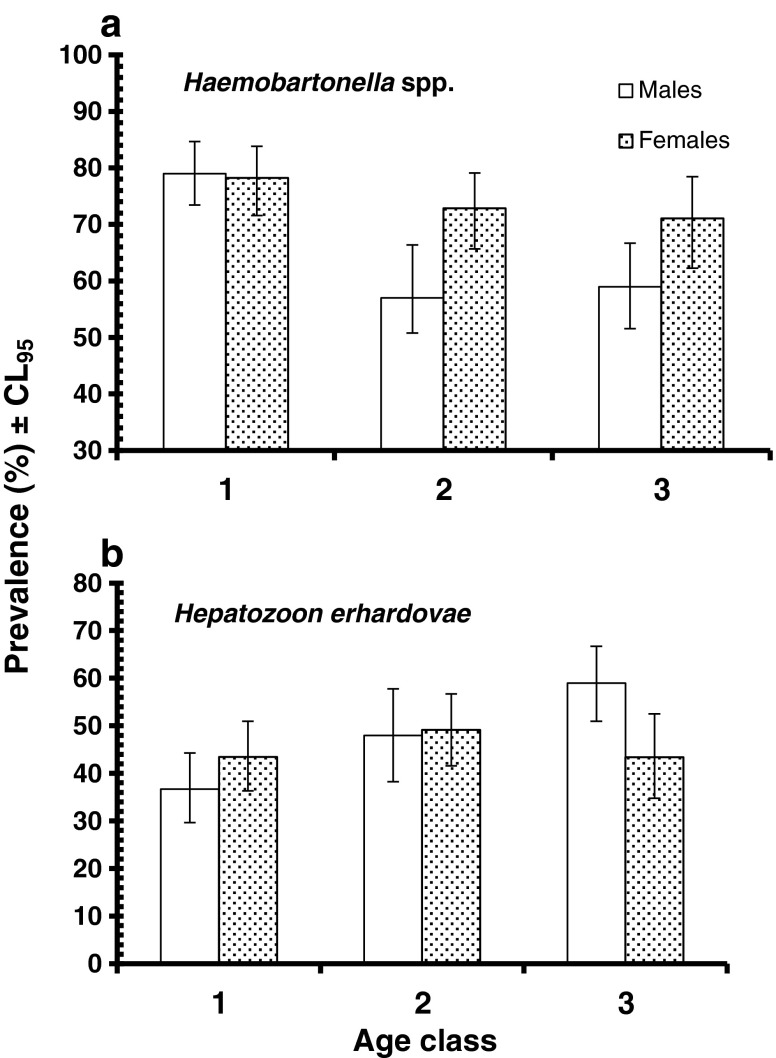
Age- and sex-dependent prevalence of *Haemobartonella* spp. (**a**) and *Hepatozoon* spp. (**b**). For statistical analysis, see text

The overall prevalence of *Haemobartonella* spp. was 68.3 % (64.42–71.91). Variation across the four surveys by site is shown in Fig. 2d, where an increase in prevalence with year of study is evident in all three sites, but this was further confounded by an interaction with sex, which is not illustrated (year × site × sex × presence/absence of *Haemobartonella* spp., *χ*_6_^2^ = 12.9, *P* = 0.044). However, *post hoc* analysis excluding SEX, revealed that there was a highly significant interaction between year × site × presence/absence of *Haemobartonella* spp. (*χ*_6_^2^ = 33.2, *P* < 0.001). Prevalence also fell with host age (Table 3) but more markedly in male compared with female voles (Fig. 4a; sex × age × presence/absence of *Haemobartonella* spp., *χ*_2_^2^ = 7.7, *P* = 0.021).

**Fig. 5:**
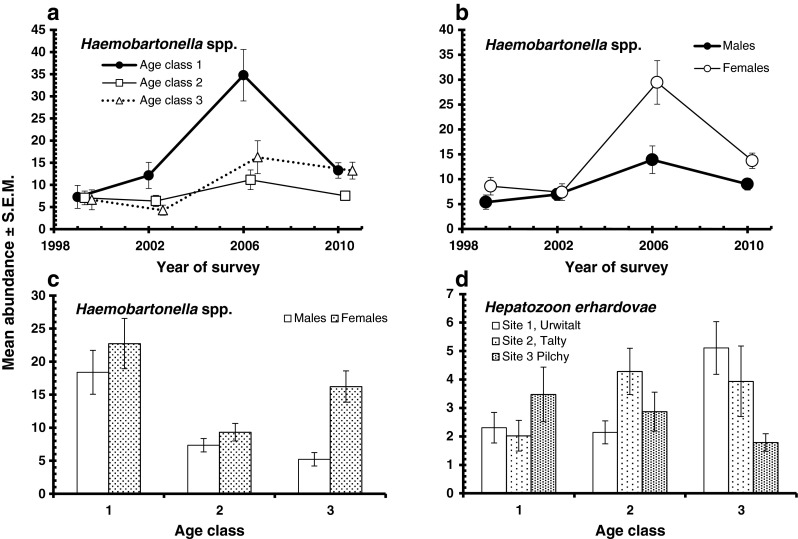
Abundance of *Haemobartonella* spp. by age class and year of survey (**a**), host sex and year of survey (**b**) and host sex and age (**c**), and of *Hepatozoon* spp. by host age and study site

The overall abundance of *Haemobartonella* spp. was 12.7 ± 0.93 iRBC/200 fields, but this varied significantly between the 4 years of the study (Table 4; GLM with negative binomial errors, main effect of year LR_3_ = 40.98 *P* < 0.0001) and the pattern of change differed between the three sites. In Urwitałt, there was relatively little change in abundance over the period, but in both Tałty and Pilchy, there were comparable peaks of abundance in 2006, followed by a return to base levels similar to those in Urwitałt by 2010 (Fig. 3d, two-way interaction year × site, LR_6_ = 23.14, *P* < 0.001). The three age classes also differed in infection level (Table 4; main effect of age, LR_2_ = 24.6, *P* < 0.0001) with the youngest age class showing a higher mean number of iRBC in all four surveys, but with a particularly pronounced peak of abundance in 2006 (Fig. 5a, two-way interaction year × age, LR_6_ = 17.54, *P* = 0.0075). Abundance also differed significantly between the sexes with a higher mean number of iRBC in females compared to males throughout (Table 4; main effect of sex, LR_1_ = 16.85, *P* < 0.00001), but the sex difference varied across the years. Females and males displayed similar levels of infection in 1999 and 2002, but in 2006 and 2010, abundance was much higher in females (Fig. 5b; two-way interaction year × sex, LR_3_ = 8.39, *P* = 0.0075). There was also a significant difference in the age effect in the two sexes, with abundance at its highest in the youngest voles, then falling in age class 2 voles and age class 3 males, but increasing again in age class 3 females (Fig. 5c; two-way interaction age × sex, LR_2_ = 10.47, *P* = 0.0053).

#### *Hepatozoon Erhardovae*

The prevalence of *H. erhardovae* was 46.8 % (42.75–50.7) overall, but there was a marked reduction with each successive year of the study (Table 3; year × presence/absence of *H. erhardovae.*, *χ*_3_^2^ = 49.3, *P* < 0.001), and this was consistent in all three sites (Fig. 2e). Prevalence also increased with host age in males, but showed a more stable pattern across age classes in females (Fig. 4b; sex × age × presence/absence of *Hepatozoon* spp., *χ*_2_^2^ = 7.8, *P* = 0.02).

The overall abundance of *H. erhardovae* was 3.1 ± 0.26 parasites/200 fields. The highest mean numbers of parasites in all three vole populations were recorded in 1999 (Table 4), after which abundance fell consistently over the following years to lows at all three sites in 2010 (Fig. 3e; GLM with negative binomial errors, main effect of YEAR, LR_3_ = 66.5, *P* < 0.00001). The rate of change in infection levels over the period varied between sites (Fig. 3e; two-way interaction, site × year, LR_6_ = 14.2, *P* = 0.026), and there was a marginal overall difference between populations with the lowest abundance value in Pilchy (Table 4; LR_2_ = 6.0, *P* = 0.05). Infection levels did not differ significantly between the sexes (LR_1_ < 0.001) nor between the age classes (Table 3, LR_2_ = 5.2, *P* = 0.073), but as can be seen from Fig. 5d, peak abundance was associated with different age classes in each of the three populations (in age class 3 at Urwitałt, age class 2 in Tałty and age class1 in Pilchy, two-way interaction, site × age LR_4_ = 14.8, *P* = 0.005).

#### *Trypanosoma Evotomys*

The overall prevalence of *T. evotomys* was 15.4 % (12.68–18.48), and this varied distinctly between bank vole populations with a lower infection rate among voles in Pilchy (Table 3; site × presence/absence *T. evotomys*, *χ*_2_^2^ = 13.5, *P* = 0.001). Statistically, prevalence showed temporal stability (Table 3 and Fig. 2f). Nevertheless, as can be seen prevalence fell quite markedly in Pilchy but not in the other two populations, creating a large discrepancy in 2006 (4 versus 24 %, in Pilchy and Urwitałt and Talty, respectively). Prevalence also increased significantly with host age (Table 3; age × presence/absence of *Trypanosoma* spp., *χ*_2_^2^ = 14.4, *P* = 0.001).

The overall abundance of *T. evotomys* was 6.6 ± 1.05 parasites/200 fields, but the data were highly over dispersed (*I* = 140.95, *D* = 0.95. *k* = 0.0317 ± 0.000006; for goodness of fit to negative binomial *χ*_5_^2^ = 13.1, *P* = 0.022). Parametric models based on transformed data failed to show normally distributed residuals and those based on negative binomial errors failed to converge so analysis was based on non-parametric tests only. The abundance of *T. evotomys* did not change significantly over the period (Table 4, Kruskal–Wallis test, *χ*_3_^2^ = 5.88, *P* = 0.12), but there was a significant difference between sites, with a higher abundance in the Urwitałt population compared to the other two sites (Table 4, *χ*_2_^2^ = 13.2, *P* = 0.001), and this was evident in all four surveys (Fig. 3f). The interaction could not be tested statistically, but Fig. 3f shows that while in the Urwitałt population relatively high levels of infection were maintained throughout, at Tałty and Pilchy, the abundance of *T. evotomys* declined with year of study. There was no difference in infection level between the two host sexes, but there was a significant age effect with a peak of abundance in age class 2 voles (Table 4, *χ*_2_^2^ = 12.76, *P* = 0.002).

### Comparison of Three Bank Vole Populations

Total species richness (*n* = 5), total haemoparasite prevalence and the dominance structure (hierarchy of prevalence of the five taxa, with *Haemobartonella* spp. most prevalent, then *H. erhardovae*, *Bartonella* spp., *T. evotomys* and *B. microti* the rarest) of the haemoparasite community (Table 3) were similar in all three bank vole populations. Differences at a local scale were observed in mean species richness and prevalence of certain parasite species (Tables 3 and 4). Overall mean species richness was the lowest at Pilchy (Table 4) and showed the lowest value at this site during three of the four surveys (Fig. 3a). The population at Pilchy was also distinct by the highest prevalence and abundance of the tick-borne *B. microti* and lowest prevalence of the flea-borne *Bartonella* spp., *H. erhardovae* and particularly *T. evotomys* (Table 3).

## Discussion

The results of this long-term field study have established convincingly that indeed, as predicted, some components of the haemoparasite community of bank voles in model boreal forests show dynamic changes with time, whilst counter to our expectations, others are associated with temporal stability, but as will be made clear below, much depends on the level of analysis. Importantly, our results also emphasise the significant role of bank voles as reservoir hosts of zoonotic VBP in this region of Eastern Europe; notably *B. grahamii* and *B. microti*. Recently, we have detected for the first time two cases of *B. microti* Jena strain infection in forestry workers in NE Poland [80]. The long-term persistence of haemoparasitic infections in rodents, as reflected in our data, implies the existence of a range of suitable vectors, and this is supported by our earlier work in the same study sites, in which we reported the very high prevalence (about 80–100 %) of ectoparasitic infestations on woodland and fallow land rodents throughout the rodent breeding season [61, 65, 66, 71, 78]. The molecular techniques used here for the identification of parasites confirmed our previous species recognition on the basis of morphometrics and host specificity [9]. Additionally, this enabled the first deposition of a part of the 18S rDNA sequence of *H. erhardovae* in GenBank and clearly confirmed the presence of two distinct *H. erhardovae* genotypes parasitising bank voles across Europe [27].

We hypothesised that extrinsic factors would have the major influence on haemoparasite communities, notably through the largely unpredictable long-term temporal effect, resulting in distinct between-year dynamics. In our earlier short-term studies, year and season of study were always the factors with the most important influence on both prevalence and abundance of haemoparasitic infections in rodents [9, 58, 81]. As populations of many rodent host species fluctuate also markedly over time, in some cases showing regular cycles over several years [26] but often without a predictable between-year pattern (i.e. see tables with relative rodent densities in [5, 9, 58, 79]), similar fluctuations can be observed in their ectoparasites/vectors [61, 78], and in consequence in VBP. On a shorter time scale, within an annual cycle, peak prevalence of *T. micr*oti infections in *M. agrestis* has been reported to follow 3 months after peak flea infestation [68]. Thus, differences in prevalence and abundance of haemoparasites between years of study are often very pronounced [58] but not easy to predict precisely, especially in terms of the magnitude of change between years. It was surprising therefore in the current work to encounter so few significant between-survey effects on measures of haemoparasite infection.

The unexpected spatiotemporal stability of the haemoparasite community of bank voles at the regional level was primarily evident in a lack of significant variation in the prevalence of haemoparasites (all species combined) and mean species richness between consecutive surveys over the 11-year period of our study. Likewise, three of the five species showed no significant variation in prevalence and two no difference in abundance between the four surveys. Moreover, as expected, we found no overall difference in the prevalence of haemoparasites (all species combined) between sites although for species richness, there was a significant difference, with bank voles from Pilchy showing a lower overall value. Only the prevalence of *T. evotomys* varied significantly between sites, although three of the five species showed significant variation in abundance between sites. Our surveys were all carried out in late summer and early autumn (August/September), which is known from earlier studies to be the period of the year with the highest prevalence and abundance of the majority of haemoparasites in wild rodents in Poland and elsewhere in Europe [9, 58, 68, 76, 79], and this was reflected in the current work in the overall high prevalence of haemoparasites (90.8 % with all species combined).

Despite the relative stability of prevalence over time at the regional level (but with the site effect and intrinsic factors all taken into account in the analyses), there were some interesting trends in particular species. For example the prevalence of *H. erhardovae* declined almost linearly between successive surveys from 64.2 % in 1999 to 35.0 % in 2010 and the lack of a significant site by year interaction showed that prevalence fell at all three sites over this period to much the same extent. A significant decline over this period was also evident in the abundance of this species. Such a consistent unidirectional change over time implies that the conditions for the survival of the parasite in question were changing and since this species is flea-transmitted, changes in flea populations are likely to have been partly responsible although the influence of other, as yet unknown factors cannot be eliminated at this stage. More complex site by year interactions were recorded for *B. microti*, whose prevalence in the earlier period rose from survey to survey and then dropped in 2010, but the increase in 2006 was primarily driven by a much higher prevalence in just one site (Pilchy). Abundance among bank voles also varied significantly between years for this species, and additionally between bank voles from the different sites despite the generally low values but with an exceptionally high value recorded for voles from Pilchy in 1999 and then consistently higher values for those from Urwitałt in the remaining years. *Haemobartonella* spp. showed no significant overall regional change with time or difference between bank voles from different sites but when examined at each site in turn, significant interactions emerged. Prevalence increased in voles at all three sites over the first three surveys, although at different rates and the relative ranking of prevalence in voles from the different sites changed in successive years. For abundance, there was a major increase in voles from two sites in 2006 but not those at Urwitałt, where abundance was considerably more stable over the whole of the 11-year period.

The fact that mean species richness remained constant (varying only from 1.69 to 1.87 between years) is consistent with the idea that in the long-term haemoparasites provide some selective pressure on host fitness since neither here, nor in any of our other studies focussing on the same five species of haemoparasites has a population average equal to 2.5 or more been recorded, even among the oldest animals. Mean species richness calculated in this study was slightly higher than noted before in bank voles in the region (1.4 in [9]), although in that study, the lower values typically associated with early spring and late autumn were included in the calculation of the overall mean. Of the 845 voles that could be directly compared in the present work only one carried five species (0.1 %) and 34 four species (4.0 %) of haemoparasites. Multispecies infections are subject to both positive and negative interactions between the individual species [75] and collectively are likely to have a negative impact on rodents making them more susceptible to predation from the range of carnivorous birds and mammals abundant in the Mazury lake district, as well as reducing their overall reproductive fitness, ability to hold territories and possibly longevity [68]. Therefore, in practical terms for relatively short-lived small mammals such as bank voles, there appears to be a population level ceiling for mean species richness of haemoparasites, and hence little variation between years. Differences between years in the derived measures that we calculated were more evident when focussing on the different host populations (sites) and in the case of certain species, rather than for the overall regional community referred to above (with sites combined in summary statistics, but nevertheless taken into account in the analyses). Only in undisturbed natural environments/habitats, comprising several study sites in the same climatic zone, where all possible transmission routes are available, can these kinds of Stable H/P interactions be detected for relatively long periods of time, disguising more dynamic underlying changes in individual host populations and for particular species of parasites, because of counter-trends that effectively neutralise one another when combined in statistical analyses.

We predicted that the effect of the intrinsic factors host age and sex would be reflected in repeatable patterns (for host age) or possibly non-significant effects (for host sex) due to the increasing risk of contracting infection with duration of survival in the case of the former and to long periods of co-evolution of the individual H/P relationships in both. Significant differences between the sexes in prevalence of haemoparasites in rodents have been reported previously (male bias for *Hepatozoon* sp. [39]) but are often confounded by interactions with other factors (season- and age-dependent sex differences in *H. erhardovae* [9] and *B. microti* [58]) and generally are rather weaker effects than the influence of extrinsic factors. Here, as predicted, there was almost no effect of host sex on our derived measures with just one exception, *Haemobartonella* (*Mycoplasma*), the abundance of which was 67 % higher in female voles compared with males. Prevalence was also higher among females although with other factors taken into account the sex effect on prevalence was not significant on its own. Prevalence was higher among female voles in age classes 2 and 3 (reflected in a significant interaction between sex and age) compared with juveniles, and a similar trend was detected with abundance. The difference in mean abundance between the sexes was always in favour of higher values among females in all four surveys, but the discrepancy was particularly marked in 2006, so it is possible that female voles were more exposed to and susceptible to infection with this parasite in that year. Although the internal, within-host environment with which haemoparasites have to contend differs between males and females due mainly to different sex hormone concentrations, with possible knock-on effects on host immunity [2, 33, 37, 63], with the exception of *Haemobartonell*a spp., this largely did not affect the haemoparasite communities in our study. Other explanations that have been proposed for sex-biased prevalence of infections include differences between the sexes in body size and occupied territories, but both of these have been used to explain higher prevalence in males rather than females [38, 67]. Female bias in parasitic infections is rarer [51], but mite infestations on bats have been attributed to greater colonial aggregation compared with the more solitary existence of males [25].

As in earlier reports, host age had a significant influence on some haemoparasite species [9, 39, 58, 76, 79], but here, two contrasting trends were observed. Consistent with our expectations, a significant age-related increase in prevalence was observed for *T. evotomys*, reflecting the increasing risk of acquiring infection with increasing age, and a trend in the same direction was evident for *H. erhardovae*, although with other factors taken into account this was not significant, and both findings are in agreement with our earlier studies in bank and common voles in Poland [9, 58]. Interestingly, the abundance of *T. evotomys* declined in the oldest animals, as reported also for prevalence by Healing [39] and by Smith et al. [68] for *T. microti* in *M. agrestis*, suggesting the development of resistance among the oldest animals in these species [1, 49]. In the case of *Bartonella* spp. and *Haemobartonella* (*Mycoplasma*), the age-related pattern was quite different, peaking in the very youngest animals and falling with host age in both prevalence and abundance. A high prevalence in the youngest voles has been found previously for *Bartonella* spp. in a common vole population [58], reported also by Welc-Falęciak et al. [79] for bank and root voles and by Turner [76] for bank voles in the UK. Juvenile naive voles must therefore be heavily exposed to infections, perhaps through greater contact with flea vectors or through vertical transmission from infected mothers to their offspring. Successful isolation of these bacteria from the foetuses of rodents (white-footed mouse and cotton rats) suggests that transmission of *Bartonella* spp. in utero occurs among natural hosts [46]. The vertical route of transmission from infected mothers is likely therefore to initiate infection, and may be more efficient than vector dependent transmission, accounting for the 50–55 % prevalence among juvenile voles in our study. Similar conclusions were made for *Mycoplasma* infections in dogs and cats, suggesting a maternal source of infection for pups [83].

In the present work, we studied bank voles living in three sites within 13 linear kilometres of one another, and yet we found some marked differences in the patterns and extent of haemoparasite infections between sites, often confounded further by site-specific temporal variation. We did not expect such differences because each of the woodland sites was essentially ecologically very similar, and we reasoned that in contrast to helminths where such differences at a local scale are known to be pronounced [12], the dependence of haemoparasites on vectors would essentially smooth out any differences. However, counter to our expectations, there were significant and consistent between-site differences. Many studies in the literature are based on sampling at one site only, and then analysed and reported as reflecting the typical pattern of parasitic infections in that region. On the basis of our results, this is clearly an inappropriate approach. To understand fully how haemoparasites relate to their host in any chosen geographical/climatic region, it is necessary to sample concurrently multiple sites, and then to search for overall patterns as well as site-specific effects. Finally, our results provide a unique data-set enabling further dissection of the influence of contributory ecological factors on the characteristic blood parasites of bank voles in boreal forests, a model habitat for Central Europe, and future analyses should reveal in greater detail and complexity the relative importance of the individual influences on haemoparasite infections in our host populations.
